# Improving Localized Radiotherapy for Glioblastoma via Small Molecule Inhibition of KIF11

**DOI:** 10.3390/cancers15123173

**Published:** 2023-06-13

**Authors:** Miranda M. Tallman, Abigail A. Zalenski, Ian Stabl, Morgan S. Schrock, Luke Kollin, Eliane de Jong, Kuntal De, Treg M. Grubb, Matthew K. Summers, Monica Venere

**Affiliations:** 1 Department of Radiation Oncology, James Cancer Hospital and Comprehensive Cancer Center, College of Medicine, The Ohio State University, Columbus, OH 43210, USA; mmontgomery527@gmail.com (M.M.T.); azalenski18@gmail.com (A.A.Z.); moschrock@gmail.com (M.S.S.); luke.kollin@osumc.edu (L.K.); dejong.24@buckeyemail.osu.edu (E.d.J.); dek@miamioh.edu (K.D.); grubbt@ccf.org (T.M.G.); matthew.summers@osumc.edu (M.K.S.); 2 Biomedical Sciences Graduate Program, The Ohio State University, Columbus, OH 43210, USA; 3 Neuroscience Graduate Program, The Ohio State University, Columbus, OH 43210, USA

**Keywords:** glioblastoma, radiotherapy, KIF11

## Abstract

**Simple Summary:**

Glioblastoma, IDH-wild type (GBM) is the most common malignant primary brain tumor. Advances in cancer therapy remain unsuccessful in the treatment of GBM patients and have not extended the median survival beyond 12–18 months with the current treatment of surgery, chemotherapy, and radiotherapy. A central issue to finding a curative treatment option is the radioresistant nature of GBM. The goal of our study was to validate the therapeutic efficacy of enriching GBM tumor cells in the phase of the cell cycle where they are most vulnerable to radiotherapy, mitosis, using a small molecule inhibitor to the mitotic kinesin, KIF11. We confirmed that KIF11 inhibition radiosensitized GBM cells and improved overall survival in preclinical mouse models of GBM. These findings offer a new therapeutic modality that can increase the efficacy of radiotherapy for GBM with the ultimate goal of improving patient outcomes.

**Abstract:**

Glioblastoma, IDH-wild type (GBM) is the most common and lethal malignant primary brain tumor. Standard of care includes surgery, radiotherapy, and chemotherapy with the DNA alkylating agent temozolomide (TMZ). Despite these intensive efforts, current GBM therapy remains mainly palliative with only modest improvement achieved in overall survival. With regards to radiotherapy, GBM is ranked as one of the most radioresistant tumor types. In this study, we wanted to investigate if enriching cells in the most radiosensitive cell cycle phase, mitosis, could improve localized radiotherapy for GBM. To achieve cell cycle arrest in mitosis we used ispinesib, a small molecule inhibitor to the mitotic kinesin, KIF11. Cell culture studies validated that ispinesib radiosensitized patient-derived GBM cells. In vivo, we validated that ispinesib increased the fraction of tumor cells arrested in mitosis as well as increased apoptosis. Critical for the translation of this approach, we validated that combination therapy with ispinesib and irradiation led to the greatest increase in survival over either monotherapy alone. Our data highlight KIF11 inhibition in combination with radiotherapy as a new combinatorial approach that reduces the overall radioresistance of GBM and which can readily be moved into clinical trials.

## 1. Introduction

Less than 10% of glioblastoma (GBM, isocitrate dehydrogenase [IDH]-wild-type) patients survive longer than 5 years and the average length of survival after diagnosis is a dismal 12 to 18 months [1,2,3,4]. Standard of care for GBM includes radiotherapy, yet we and others have shown that GBM cells are refractory to this treatment, which contributes to tumor recurrence [5,6,7,8,9]. There is therefore a critical need to identify treatment modalities that can improve the efficacy of localized radiotherapy for GBM.

GBM is an inherently highly proliferative and mitotically active tumor and we and others have previously shown that perturbing mitosis is an effective means of limiting GBM tumor growth [10,11,12,13,14,15]. Specifically, we reported that the mitotic kinesin KIF11 (kinesin family member 11), required for bipolar spindle formation during mitosis, is elevated in GBM and portends poor prognosis [14]. We also demonstrated that the survival of mice bearing orthotopic GBM was prolonged using ispinesib, a small molecule inhibitor to KIF11 [14]. Notably, KIF11 inhibitors will arrest cells in mitosis, a phase of the cell cycle when cells are particularly vulnerable to radiotherapy [16,17,18,19]. Early studies indicated that this increased sensitivity to irradiation was linked to the compacted chromatin within mitosis being more vulnerable to DNA strand breaks, versus the dispersed chromatin of interphase cells [19]. More recent work has elucidated that, unlike the other phases of the cell cycle, DNA breaks that occur in mitosis do not trigger a cell cycle arrest unless the breaks are at telomeres or centromeres [20,21,22]. This leads to an overall increased sensitivity to DNA damage in mitosis [19,23,24]. The DNA lesions can be marked as damaged in mitosis and repaired in G1, but the increased chromosomal instability caused by mitotic progression in the presence of DNA breaks can also lead to an increase in cell death [20,23,24,25,26,27,28,29,30]. Hence, enriching GBM cells in mitosis prior to radiotherapy could serve to increase the level of tumor cell death. However, it is unknown if targeting KIF11 will radiosensitize GBM.

The goal of our study was to fill this gap by testing the hypothesis that KIF11 inhibition would serve to radiosensitize GBM by enriching the fraction of GBM cells within the radio-sensitive mitotic phase of the cell cycle. We were able to confirm KIF11 inhibition as a radiosensitizer using in vitro clonogenic assays. Our in vivo studies highlighted an increase in mitotic index following ispinesib treatment. Importantly, we confirmed that combinatorial treatment with ispinesib and radiotherapy significantly improved overall survival in our preclinical models. Taken together, our findings highlight enrichment in mitosis as a therapeutic paradigm that can enhance the efficacy of localized radiotherapy for GBM.

## 2. Materials and Methods

### 2.1. Cells and Cell Culture

All cells were obtained as de-identified specimens that were initially acquired as primary human brain tumor patient specimens in accordance with appropriate, approved Institutional Review Board (IRB) protocols. Of these cells, 3691 was a kind gift from Dr. Jeremy Rich (University of Pittsburgh), 1016 was a kind gift from Dr. Anita Hjelmeland, and NU757 was obtained from the Northwestern University Nervous System Tumor Bank.

Cells were cultured at 37 °C at 5% CO_2_ in Neurobasal media (minus phenol red; Gibco, Grand Island, NY, USA) supplemented with B27 (minus Vitamin A; Gibco), human fibroblast growth factor-2 (10 ng/mL; Miltenyi Biotec, Bergisch Gladbach, Germany), human epidermal growth factor (10 ng/mL; Miltenyi Biotec), L-glutamine (2 mM; Gibco), sodium pyruvate (1 mM; Gibco), and penicillin/streptomycin (100 I.U./mL/100 μg/mL; Gibco). Cells plated adherently were on Geltrex LDEV-Free hESC-Qualified, Reduced Growth Factor Basement Membrane Matrix (Gibco), whereas in vivo studies were performed with cells grown in suspension as tumorspheres before dissociation and cell counting prior to implantation. TrypLE Express Enzyme was used to obtain single cell suspensions (no phenol red; Gibco). Mycoplasma testing was performed quarterly (Mycoplasma Detection Kit; Southern Biotech, Birmingham, AL, USA) and cell line verification was performed annually (microsatellite genotyping; Ohio State University Comprehensive Cancer Center Genomics Shared Resource).

### 2.2. Animals and In Vivo Studies

All animal studies described were approved by the Ohio State University Institutional Animal Care and Use Committee and conducted in accordance with the NIH Guide for the Care and Use of Laboratory Animals. Male and female athymic Nu/Nu mice were used for all studies and were obtained from the Ohio State University Comprehensive Cancer Center Target Validation Shared Resource. Cells at 1 × 10^4^ were injected intracranially in a total volume of 2 μL Neurobasal media (no supplements) 2 mm into the right lateral part of bregma, and at a depth of 2.5 mm from the dura, in mice 6–8 weeks old. All mice were monitored daily for early removal criteria including neurological impairments and/or a drop in weight of more than 20% of their original weight. For single treatment studies, designed to compare mitotic index temporally and between delivery methods, tumor burden was established for 28 days and, then, mice were randomized into one of three treatment groups: vehicle, ispinesib (10 mg/kg, intraperitoneal), or ispinesib (10 mg/kg, intravenous via the tail vein), with mice from each group sacrificed 6 or 12 h after treatment. For full treatment and survival studies, mice were randomized into one of four treatment groups seven days after implantation: vehicle, ispinesib (10 mg/kg, intravenous), irradiation (2.5 Gy), or ispinesib and irradiation. Initiation of treatment was based on previous studies whereby tumor burden was known to have been established 7 days post-implantation for 3691 and 14 days post-implantation for 1016. Ispinesib or vehicle treatments were given once a week for four weeks (7, 14, 21, and 28 days after intracranial injection of 3691 and 14, 21, 28, and 35 for 1016). Irradiation was given to the tumor-bearing hemisphere 6 h after vehicle or ispinesib injections using the Small Animal Radiation Research Platform (SARRP; Xstrahl Medical and Life Sciences) for targeted dose delivery. All mice in the full treatment study were sacrificed 6 h after the irradiation was given to mice in those cohorts. For the survival study, mice were sacrificed upon meeting early removal criteria.

### 2.3. Small Molecule Inhibitor

Ispinesib was obtained from Selleck Chemicals (#S1452). For in vitro experiments, stock solutions of ispinesib were made in DMSO. Working concentrations were made immediately before use and diluted in cell media. DMSO served as the vehicle control. For in vivo work, working dilutions of ispinesib were made immediately before use in EtOH followed by Tween-80, and then sterile water at a ratio of 20:25:77.5, respectively. The EtOH, Tween-80, and sterile water mixture served as the vehicle control for in vivo studies.

### 2.4. Colony Formation Assays

Cells were plated at 250 cells per well onto Geltrex treated 6-well plates. The next day, cells were treated with ispinesib at 0.35 nM or with vehicle control (DMSO), and immediately left unirradiated (0 Gy) or irradiated with 1, 2, or 3 Gy. Irradiation was performed using a GammaCell 40 Irradiator (Best Theratronics). Sham irradiated control plates (0 Gy) were transported to the radiation facility, but not exposed. Media was changed 24 h later. Ten days post-treatment, cells were washed before being fixed and stained with a 0.5% crystal violet solution. Plates were imaged on the LI-COR Odyssey near infrared imaging system and analyzed via an ImageJ macro, which counts individual colonies, allowing for unbiased quantification.

### 2.5. Hematoxylin and Eosin Staining

Mice were perfused (1x PBS followed by 4% PFA) and tumor-bearing brains were harvested, fixed in 4% PFA overnight at 4 °C, sucrose sunk at 4 °C (30% sucrose solution), and then embedded in OCT compound. Sections of 10 µm were mounted onto slides (Superfrost Plus Microscope Slides; Fisherbrand, Pittsburgh, PA, USA) and stored at −20 °C till further processing. Sections were brought to room temperature for 30 min and then desiccated until dry (about 15 min). Sections were stained with hematoxylin (2 min) and eosin (20 s), followed by treatments with EtOH (20 s, three times) and xylenes (1 min, two times). Coverslips were mounted using Fluoromount-G Mounting Medium (Southern Biotech). Sections were imaged on an EVOS M7000 (AMF7000 Invitrogen, Software Version 2.0.2094.0) using the 10x objective.

### 2.6. Immunocytochemistry

Sections, as above, were warmed to room temperature for 2 h. Sections were then post-fixed with 4% PFA for 15 min, washed three times in 1x PBS, then blocked at room temperature for 1 h in 10% (*w*/*v*) BSA (for anti-cl-Caspase-3) or 10% goat serum (for anti-pH3Ser10) in PBS-Triton X-100 (0.2% *v*/*v*). After the block, sections were immunolabeled with anti-cleaved-Caspase-3 (cl-Caspase-3; 1:400; Cell Signaling 9664) or anti-phospho-Histone H3 Serine 10 (pH3S10; 1:1000; Cell Signaling 9706) overnight at 4 °C in a humidified chamber. The next day, slides were washed three times in PBS-Triton X-100 (0.2% *v*/*v*) followed by secondary detection with Alexa Fluor 594 (Invitrogen, Waltham, MA, USA) for 2 h at room temperature. Nuclei were counterstained with Hoechst. Coverslips were mounted using Fluoromount-G Mounting Medium (Southern Biotech, Birmingham, AL, USA). Images were acquired using EVOS M7000 (AMF7000 Invitrogen, Software Version 2.0.2094.0) and six images were taken per section (three random areas of the tumor rim and three random areas of the tumor core).

### 2.7. Image Analysis

Images were run through ImageJ (1.53f51) macros based on the marker. For pH3S10, we counted Hoechst-stained nuclei, and then calculated the percent of all cells that were positive for pH3S10. Cl-Caspase-3 was analyzed by taking the mean pixel intensity of the image.

### 2.8. Statistical Analysis

Statistical analyses were conducted using GraphPad Prism 9.4.1, unless otherwise stated. The statistical test used for each experiment is listed within the corresponding figure legend. For the colony formation assays, three biological repeats were performed for each specimen and each biological replicate included three technical replicates. For immunocytochemistry, tumors from three separate mice per condition were evaluated with six images taken per tumor for a total of eighteen separate images evaluated per condition.

## 3. Results

### 3.1. KIF11 Inhibition Radiosensitized Patient-Derived GBM Cells In Vitro

**Figure 1:**
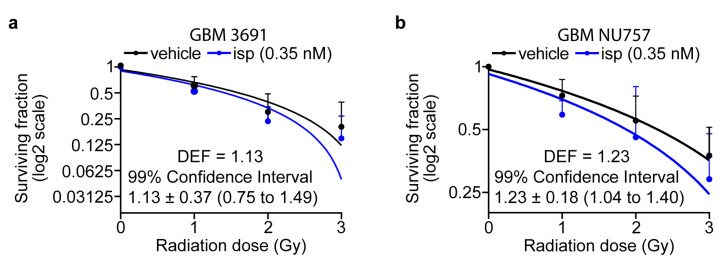
KIF11 inhibition combined with irradiation increased the radiosensitivity of GBM cells in vitro. (**a**) GBM 3691 and (**b**) GBM NU757 were treated with vehicle (DMSO) or 0.35 nM ispinesib (isp) and then irradiated (0–3 Gy). Colonies per well were normalized to 0 Gy and linear regression was used to model the effect of radiation on survival. Vehicle (black line) and ispinesib (blue line) data were graphed on log2 scale. n = 3 biological replicates per GBM specimen with n = 3 technical replicates per biological repeat. Dose enhancement factors (DEFs) were calculated by comparing doses at which the surviving fraction was 0.5 and the 99% confidence interval showed a DEF of above 1. Error bars represent standard deviation.

To begin to investigate if KIF11 inhibition was capable of radiosensitizing GBM cells, we utilized clonogenic assays to quantify reproductive cell survival after irradiation as this approach is associated with the clinical response of a tumor to radiotherapy [31,32,33,34]. GBM 3691 and GBM NU757 were treated with 0.35 nM ispinesib, a concentration that did not induce excessive cell death as a single treatment, and were then exposed to 0–3 Gy of irradiation. Clonogenic survival was reduced for both GBM specimens, with a resulting dose enhancement factor (DEF; DEF at surviving fraction 0.5 with a DEF greater than 1 indicating a synergistic effect) of 1.13 for GBM 3691 and 1.23 for GBM NU757 (Figure 1a,b). These data indicate that KIF11 inhibition via ispinesib prior to irradiation radiosensitized GBM cells.

### 3.2. The Mitotic Index and Level of Apoptosis Were Increased in Tumors following a Single Treatment with Ispinesib

**Figure 2:**
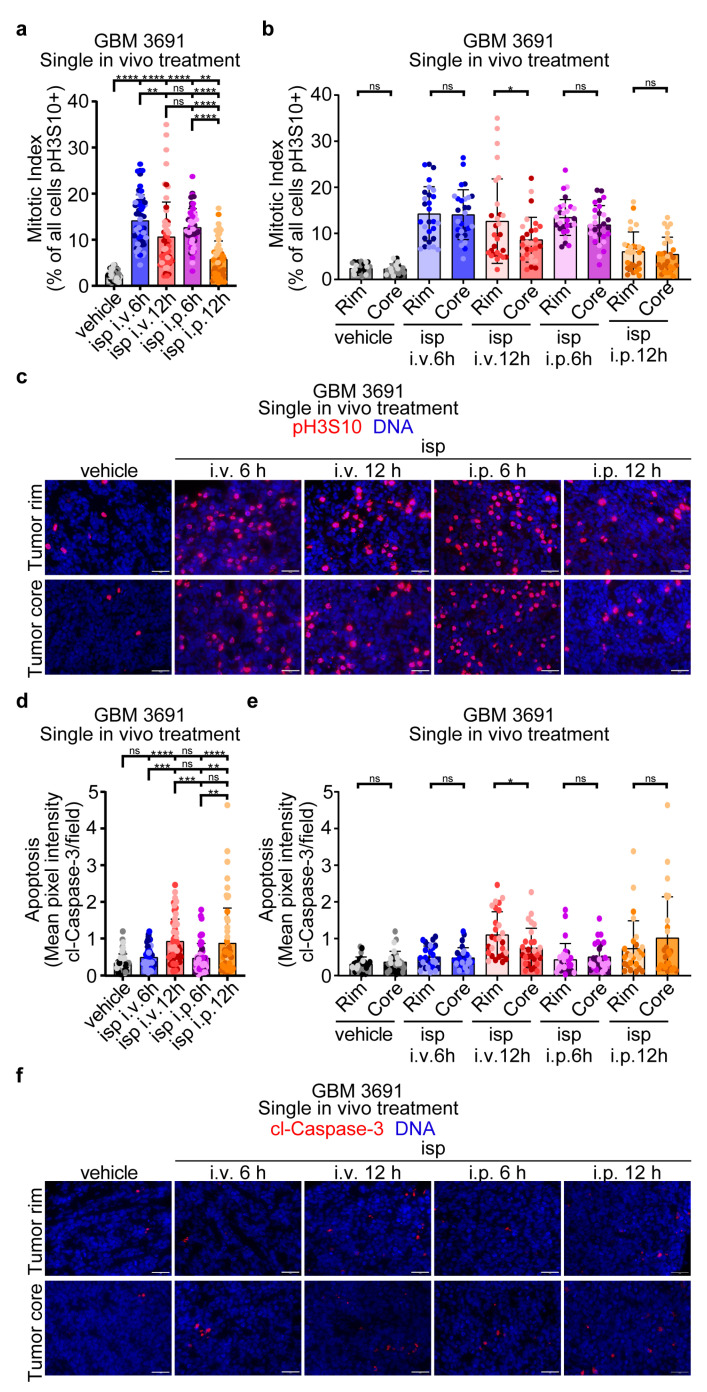
Single in vivo treatment with ispinesib increased the mitotic index and apoptosis of tumor cells. (**a**,**b**) Tumor-bearing mice were treated with vehicle or a single dose of ispinesib (isp), given intravenously (i.v.) or intraperitoneally (i.p.), and brains were harvested 6 or 12 h later. Tumor-bearing brains were sectioned and immunolabeled with anti-pH3S10 and DNA was counter-stained with Hoechst. The percentage of pH3S10-positive tumor cells was calculated for each condition. (**c**) Representative images of mitotically arrested tumor cells in each condition. (**d**,**e**) Tumor sections were immunolabeled with anti-cl-Caspase-3 and DNA was counter-stained with Hoechst. The mean pixel intensity for cl-Caspase-3 per field was measured for each condition. (**f**) Representative images of apoptotic tumor cells in each condition. Tumors from three separate mice per condition were evaluated with six images taken per tumor (three at the tumor rim and three at the tumor core) for a total of eighteen separate images evaluated per condition. Each dot within the bar graphs represents the data from an individual image and the three different color shades each represents one of the three tumors evaluated. Data were analyzed in (**a**) and (**d**) by a one-way ANOVA with a Tukey’s multiple comparison test and in (**b**) and (**e**) by Student’s *t*-test. Error bars represent standard deviation. ns, no significance; *, *p* < 0.05; **, *p* < 0.01; ***, *p* < 0.001; ****, *p* < 0.0001.

Having established KIF11 inhibition as an efficient approach to radiosensitize GBM cells in vitro, we then wanted to explore the in vivo efficacy of combination therapy. As a first step, we wanted to establish the drug delivery method and timing post-drug administration that would result in the greatest fraction of tumor cells arrested in mitosis and hence most vulnerable to irradiation. We previously found that repeated in vivo dosing of ispinesib at 10 mg/kg, given intraperitoneally (i.p.), was well tolerated, and so we chose this concentration for both i.p. and intravenous (i.v.) drug administration [14]. Mice bearing orthotopic tumors were given a single dose of vehicle or ispinesib 28 days post tumor cell implantation which, based on prior studies, is a time point with well-established tumor burden but prior to mice reaching early removal criteria [7]. Tumor-bearing brains were collected at 6 h and 12 h post-drug and evaluated for changes in the mitotic index via immunofluorescence to the mitotic marker pH3S10 (Figure 2a). Both i.v. and i.p. drug delivery, at both time points, resulted in increased mitotic indexes over the vehicle, with i.p. at 12 h having the least significance. Between i.v. and i.p. administration, the mitotic index was not statistically different between i.v. 6 h and 12 h and i.p. 6 h, but both i.v. timepoints had significantly higher mitotic indexes than the 12 h i.p. timepoint. For both i.v. and i.p. drug delivery, the earlier 6 h timepoint resulted in a significantly higher mitotic index over the later 12 h timepoint. As previous reports indicated that ispinesib concentrations were higher in the tumor core versus tumor rim, we wanted to further analyze our data to compare for differential mitotic arrest upon KIF11 inhibition between the tumor rim and the tumor core for the different delivery methods and time points (Figure 2b,c) [35]. Only the i.v. 12 h cohort had a significant difference in the mitotic index between the rim and the core. Overall, these data indicate that, despite potential differences in drug concentration across the bulk tumor, there are sufficient levels of ispinesib for target engagement and resulting mitotic arrest.

To assess if even a single treatment of ispinesib can impact tumor cell viability, we evaluated for changes in apoptosis, via immunofluorescence, to the apoptotic marker cleaved-Caspase-3 (cl-Caspase-3) for both the whole tumor, and comparing the tumor rim to the tumor core (Figure 2d–f). Interestingly, although the mitotic index was higher for both i.v. and i.p. at the 6 h timepoint, apoptosis was highest at the 12 h timepoint for both delivery methods, potentially indicating that tumor cell death increases as more cells attempt to transit into mitosis in the presence of the drug (Figure 2d). For the tumor rim and tumor core, akin to the mitotic index, only the i.v. 12 h condition had a significant difference, albeit that the overall level of apoptosis, as measured by cl-Caspase-3, was very low in all treatment groups (Figure 2e,f). Given the maximal response in mitotic index at 6 h post i.v. administration, we chose this delivery method and timepoint post-drug to give radiotherapy for further in vivo studies. Taken together, these data indicate efficient KIF11 inhibition by ispinesib via different delivery methods and at different timepoints.

### 3.3. Repeated In Vivo Treatment with Ispinesib, with and without Radiotherapy, Led to Increased Mitotic Indexes and Tumor Cell Death

**Figure 3:**
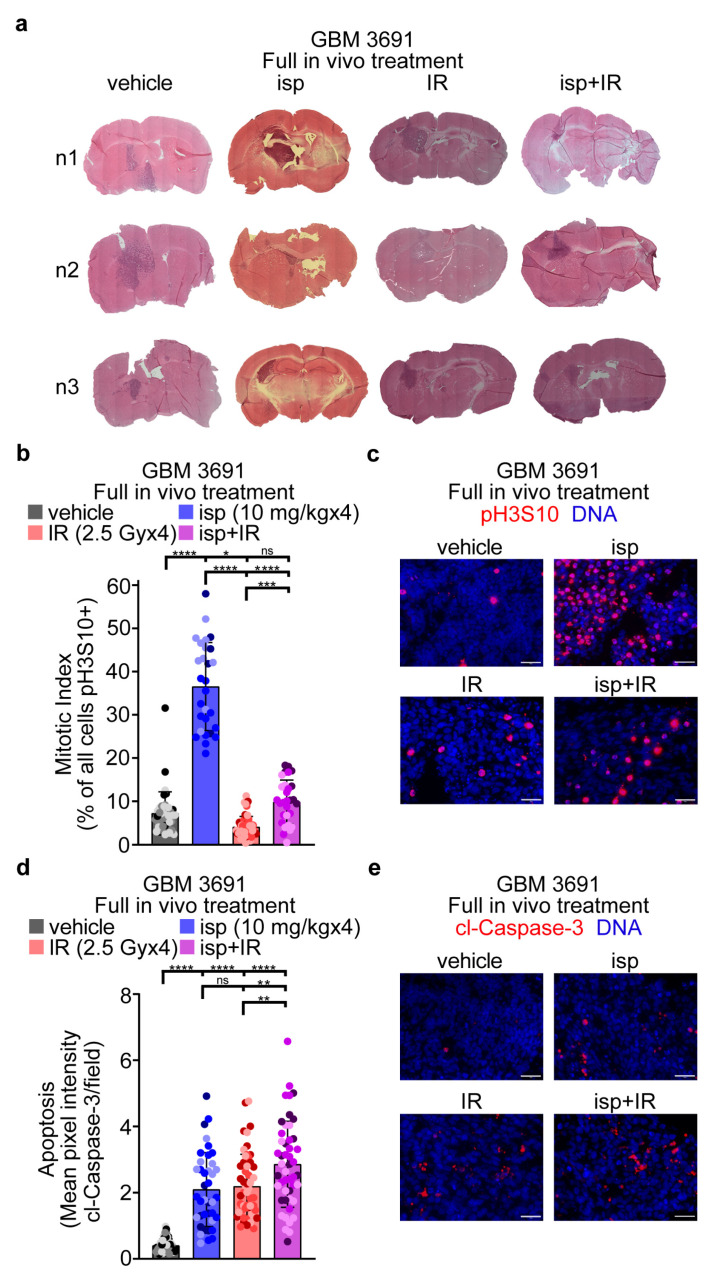
Multiple in vivo treatments with ispinesib increased the mitotic index and apoptosis of tumor cells. (**a**) Representative hematoxylin and eosin images of tumor-bearing brains following the full treatment timecourse for each cohort. (**b**) Tumor-bearing brains were sectioned and immunolabeled with anti-pH3S10 and DNA was counter-stained with Hoechst. The percentage of pH3S10-positive tumor cells was calculated for each condition. (**c**) Representative images of mitotically arrested tumor cells in each condition. (**d**) Tumor sections were immunolabeled with anti-cl-Caspase-3 and DNA was counter-stained with Hoechst. The mean pixel intensity for cl-Caspase-3 per field was measured for each condition. (**e**) Representative images of apoptotic tumor cells in each condition. Tumors from three separate mice per condition were evaluated with six images taken per tumor for a total of eighteen separate images evaluated per condition. Each dot within the bar graphs represents the data from an individual image and the three different color shades each represents one of the three tumors evaluated. Data were analyzed in (**b**) and (**d**) by a one-way ANOVA with a Tukey’s multiple comparison test. Error bars represent standard deviation. ns, no significance; *, *p* < 0.05; **, *p* < 0.01; ***, *p* < 0.001; ****, *p* < 0.0001.

Having established the optimal delivery method and time post-administration for mitotic enrichment following ispinesib treatment, we next wanted to evaluate mitosis and apopotosis in tumors exposed to multiple drug treatments, as well as to combinatorial treatment with radiotherapy. We had four cohorts: vehicle, ispinesib (10 mg/kg), radiotherapy (2.5 Gy), or ispinesib and radiotherapy. For our treatment paradigm, we gave ispinesib or vehicle weekly for 4 weeks and radiotherapy 6 h following the administration of ispinesib or vehicle. The treatment started 7 days post tumor cell inoculation and tumors for all cohorts were harvested 6 h after the final administration of radiotherapy. Hematoxylin and eosin staining confirmed tumor burden for all treatment groups at time of harvest (Figure 3a). We next evaluted mitotic index by pH3S10 (Figure 3b,c) and apoptosis by cl-Caspase-3 (Figure 3d,e). Multiple treatments with ispinesib led to the greatest increase in mitotic index over vehicle (Figure 3b), whereas all treatment groups led to an increased level of apoptosis over the control (Figure 3d). Interestingly, the combination group had a lower mitotic index in comparison to ispinesib as a monotherapy, but had a significantly higher level of apoptosis over all treatment groups. The lower mitotic index in the combination group could indicate that more mitotic cells have died following irradiation, hence resulting in an overall decrease in mitotic index, but more refined temporal studies would be required to confirm this.

### 3.4. Combination Treatment with Ispinesib and Radiotherapy Improved Survival in Preclinical Models of GBM

**Figure 4:**
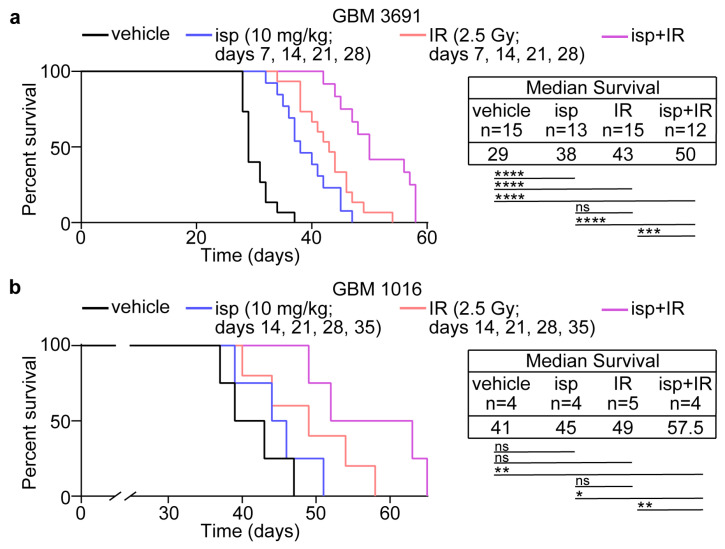
Combination treatment with ispinesib and radiothearpy increased survival in orthotopic preclinical mouse models of GBM. (**a**) GBM 3691 and (**b**) GBM 1016 orthotopic tumor bearing mice were treated with vehicle, ispinesib (isp; 10 mg/kg), irradiation (IR, 2.5 Gy) or ispinesib and IR (isp+IR) on the indicated days. Kaplan-Meier survival curves were generated for vehicle (black line), ispinesib (blue line), IR (red line), and isp+IR (purple line). The median survival and number of mice per group for each condition is indicated. Data were analyzed via independent log-rank (Mantel-Cox) tests between groups with a Bonferroni’s post-hoc multiple comparison test. ns, no significance; *, *p* < 0.05; **, *p* < 0.01; ***, *p* < 0.001; ****, *p* < 0.0001.

Given the positive in vitro data showing the radiosensitization of GBM cells via ispinesib, along with the in vivo data indicating an increase in cell death with the combination, we next wanted to evaluate if the combination treatment would provide a survival advantage. We had the same four cohorts and treatment schedule described above (i.e., vehicle, ispinesib (10 mg/kg), radiotherapy (2.5 Gy), or ispines*i*b and radiotherapy given every 7 days for 4 weeks with radiotherapy given 6 h post-ispinesib). The mice were then monitored for overall survival following cessation of treatments. We used both GBM 3691, which was used in previous in vivo mitotic index and apoptosis studies, as well as GBM 1016. For both patient-derived orthotopic models, the combinatorial therapy led to a significant increase in median survival in comparison to ispinesib or irradiation as a monotherapy as well as the vehicle cohort (Figure 4a,b). These data highlight that enriching GBM tumor cells in a radiosensitive cell cycle phase can lead to increased tumor cell death and improved survival.

## 4. Discussion

Given the inherent radioresistant nature of GBM, there have been numerous efforts to identify radiosensitizers that would serve to improve the overall efficacy of radiotherapy [36,37,38,39]. In our studies, we sought to evaluate if the enrichment of GBM cells in mitosis, using an inhibitor to the mitotic kinesin KIF11, could increase overall tumor cell death due to the increased sensitivity of mitotic cells to irradiation [16,17,18,19]. Indeed, our in vitro clonogenic assays confirmed the radiosensitization of GBM cells when pretreated with the KIF11 inhibitor ispinesib and then irradiated. We also confirmed mitotic enrichment in orthotopic preclinical mouse models of GBM, that was concomitant with an increase in cell death when tumors were also treated with radiotherapy. However, more in-depth temporal studies would serve to further strengthen the in vivo link between an increase in mitotic index and an increase in mitotic cell death following radiotherapy. Of key importance for translation, the combination therapy was able to extend survival in these mouse models.

The approach of using a KIF11 inhibitor to enrich tumor cells in mitosis prior to radiotherapy has strong rationale. However, to date, no KIF11 inhibitors have received FDA approval. This is despite the development of dozens of inhibitors with varying mechanisms of action for inhibition [40,41]. Ispinesib was the first KIF11 inhibitor to enter clinical trials and was reported to be well tolerated, but a lack of tumor response for ispinesib, and the other inhibitors that made it into clinical trials, has left the field with an overall disappointing outlook for clinical translation of KIF11 inhibitors. However, most of these studies were focused on KIF11 inhibition as a targeted, antiproliferative approach. Hence, many trials used the KIF11 inhibitor as a monotherapy. Combinatorial studies were also performed with a variety of chemotherapeutics, but none incorporated radiotherapy. Our approach of using KIF11, not only as an anti-proliferation strategy but also as a radiosensitizer, may therefore provide a new approach to achieving more positive clinical outcomes for KIF11 inhibition.

Should KIF11 inhibition plus radiotherapy move forward for GBM, which inhibitor to use and the design of the treatment schedule would be critical factors to consider. We used ispinesib in these studies based on our prior, promising work with this drug as a monotherapy for GBM [14]. Our current studies focused on human GBM models whereby we saw pronounced target engagement, as indicated by an increase in the mitotic index, following just a single dose of ispinesib. Most importantly, the combination with radiotherapy improved overall survival using multiple human GBM patient cell lines. Recent studies have reported a drug efflux of ispinesib by GBM cells and demonstrated that inhibition of the efflux pumps, in combination with ispinesib, improved efficacy in rodent and human models of GBM [35]. Although it is unknown if drug efflux is at play in our models, the combination of ispinesib and radiotherapy produced a significant impact on orthotopic tumors. For the dosing schedule, we chose a very conservative schedule for our studies, giving treatment only once a week. This treatment design nonetheless led to an overall increase in survival with the combination, demonstrating the utility of this strategy. Given that mice did succumb to tumor burden upon cessation of treatment, however, the efficacy of additional ispinesib plus radiotherapy cycles could be evaluated. Alternatively, the use of KIF11 inhibitors with a longer half-life, such as ARRY-520 with a half-life of more than 90 h, versus 16 h for ispinesib, could allow for a more frequent radiotherapy schedule to capitalize on the continued enrichment of cells in mitosis [42,43,44]. More frequent combinatorial radiotherapy could also be achieved with 4SC-205, which is an oral KIF11 inhibitor that can be administered daily [45]. Overall, our findings with ispinesib lay the foundation for future studies that could explore repeated and extended dosing of both KIF11 inhibition and radiotherapy to potentially achieve even great tumor cell death and further extension of survival if not, ideally, the full eradication of tumor burden.

## 5. Conclusions

Taken together, our work highlights a novel treatment approach for GBM that capitalizes on the radiosensitivity of cells in the mitotic phase of the cell cycle. Our work focuses on achieving this enrichment in mitosis via the inhibition of the mitotic kinesin, KIF11, but there are numerous small molecule inhibitors developed or in development for other mitotic regulators that could also be combined with radiotherapy and tested in the context of GBM. With no curative treatment options for this devastating tumor, this approach can be further explored to achieve better survival outcomes for GBM patients.

## Data Availability

No new data were created or analyzed in this study. Data sharing is not applicable to this article.
